# ZnO Hexagonal Nano- and Microplates Modified with Nanomaterials as a Gas-Sensitive Material for DMS Detection—Extended Studies

**DOI:** 10.3390/s24175690

**Published:** 2024-09-01

**Authors:** Patrycja Suchorska-Woźniak, Helena Teterycz

**Affiliations:** Faculty of Electronics, Photonics and Microsystems, Wrocław University of Science and Technology, Wybrzeże Wyspiańskiego 27, 50-370 Wrocław, Poland; patrycja.suchorska-wozniak@pwr.edu.pl

**Keywords:** resistive gas sensor, ZnO, dimethyl sulphide, gold nanoparticles, sepiolite, nanoplates, halitosis

## Abstract

The detection of dimethyl sulphide (DMS) at levels between ppb and ppm is a significant area of research due to the necessity of monitoring the presence of this gas in a variety of environments. These include environmental protection, industrial safety and medical diagnostics. Issues related to certain uncertainties concerning the influence of high humidity on DMS measurements with resistive gas sensors, e.g., in the detection of this marker in exhaled air, of the still unsatisfactory lower detection limit of DMS are the subject of intensive research. This paper presents the results of modifying the composition of the ZnO-based sensor layer to develop a DMS sensor with higher sensitivity and lower detection limit (LOD). Improved performance was achieved by using ZnO in the form of hexagonal nano- and microplates doped with gold nanoparticles (0.75 wt.%) and by using a well-proven sepiolite-based passive filter. The modification of the layer composition with respect to the authors’ previous studies contributed to the development of a sensor that is highly sensitive to 1 ppm DMS (S = 11.4) and achieves an LOD of up to 406 ppb, despite the presence of a high water vapour content (90% RH) in the analysed atmosphere.

## 1. Introduction

Dimethyl sulphide (DMS) is one of the volatile organic sulphur compounds (VSCs); it is present in large quantities in the atmosphere and plays an important role in the global sulphur cycle [1]. According to studies, about 40% of the total sulphur in the atmosphere comes from natural emissions, and the ocean is one of the main natural sources of it [2]. In addition, it is present in the aroma produced when certain vegetables are cooked, particularly cabbage, beetroot and seafood [3], and is widely used in the petrochemical, fuel and food industries as a food-flavouring additive (e.g., to improve the aroma of beer) and as an ingredient in perfumes [4]. Dimethyl sulphide is also a biomarker, a product of metabolic reactions taking place in the human body, and its concentration in exhaled air increases as a result of disorders in the functioning of, among other things, the liver. Along with hydrogen sulphide, this marker is responsible for the occurrence of halitosis [5,6,7]. The association of metabolic disorders with a specific composition of exhaled air has also contributed to the development of sensors and electronic noses, with detection levels of DMS and other biomarkers, as opposed to detection in industry being in the ppb range [8,9,10]. Therefore, continuous monitoring of DMS from ppb to ppm levels is necessary for environmental protection, production safety and also medical diagnosis.

Common detection methods for DMS include gas chromatography (GC), gas chromatography–mass spectrometry (GC-MS), gas chromatography–flame photometric detector (GC-FPD) [11,12], proton transfer reaction–mass spectrometry (PTR-MS) [13], atmospheric pressure chemical ionisation mass spectrometry (AP-CIMS) [14], tunable laser absorption spectroscopy (TLAS) [15], ion molecule reaction mass spectrometry (IMR-MS) [16] and various types of sensors [8,15,17,18,19,20,21,22,23,24]. Depending on the area in which it is applied, DMS detection has limitations, including unsatisfactory lower detection limits starting at ppb, sensor poisoning and the need for sample concentration in some methods. These limitations mean that, e.g., the detection of low concentrations of DMS by gas sensors is not as widely reported in the literature as other gases. In addition, the humidity aspect of the analysed atmosphere is often not considered.

Of the numerous detection methods presented, the requirements for the detection of volatile sulphur compounds, including dimethyl sulphide, can be met by chemical resistive gas sensors based on metal oxide semiconductors (MOSs) [25]. They offer a significantly cheaper and simpler alternative to techniques such as gas chromatography or mass spectrometry. In addition, compared to other types of sensors, their important advantages include a relatively simple operating principle, the possibility of high sensitivity (S), compact size, simple design, fast response time and low cost and energy consumption [26,27]. The MOS sensor is the most commonly used sensor type in electronic nose systems [28]. Furthermore, of particular note is the intensive development of sensors in which the active layers are made of nanomaterials [29]. Due to the fact that in this type of sensor, the reactions occurring on the surface of the gas-sensitive layer are mainly crucial during sensing, nanomaterials in sensing are desirable due to the high surface to volume ratio of the structures obtained (nanowires, nanoplates, etc.), the possibility to control the shape and dimension of the structures through the proper selection of the parameters of the sensor, the almost monocrystalline structure of nanoparticles and the easy coupling of chemical and physical properties, resulting in even more sensitive sensors with a lower detection limit (LOD) and better selectivity and stability [30].

Y. Li et al. [20] described a sensor using Co_3_O_4_ (cobalt(II, III) oxide) nanosheet-built hollow spheres containing ultrafine necked grains as the active material. The use of this p-type semiconductor oxide allowed the sensor to achieve high sensitivity to 125 ppm DMS (S = 3.0). Jang et al. [23] used flexible chemoresistors based on SnO_2_ (tin dioxide) nanosheets functionalised with Pt nanoparticles, obtaining a sensor sensitive as low as 1 ppm DMS (S = 4.84). M. Krawczyk et al. [31] presented the results of DMS detection using β-Ga_2_O_3_ (β-gallium oxide) modified with gold nanoparticles as the active layer. It was observed that the presence of Au nanoparticles improves the sensitivity of the sensor (S = 2.0 to 16 ppm DMS) and, in addition, a different response mechanism was observed than in the case of the unmodified β-Ga_2_O_3_ layer.

Key aspects to be considered in the development of MOS gas sensors for DMS detection are the modification of the active layer composition (e.g., by appropriate selection of the material and its microstructure), the use of modifying dopants (activators), the selection of a filter with a suitable microstructural structure and chemical composition and affinity for the detected compounds. Various improvements are possible due to the huge number of modification possibilities to both the receptor and actuator parts. This speaks to the enormous potential of this type of sensor.

The use of zinc oxide in VSC detection deserves special attention. This n-type semiconductor has a number of properties that cause this compound to be used in electronics and photonics. These include, among others, a wide band gap (3.37 eV), low fabrication cost, chemical and thermal stability, high electromechanical coupling coefficient and high exciton binding energy [32,33]. In addition, a very important advantage of this oxide, desirable for sensing techniques, is the possibility of obtaining different morphological forms, by appropriate selection of the synthesis method and parameters [34,35,36]. A preliminary study of improved sensor sensitivity to dimethyl sulphide using ZnO was presented by the authors in an earlier article [21], where ZnO grains doped with Au nanoparticles with a sepiolite filter were proposed. It was observed that doping zinc oxide with gold nanoparticles increased the sensitivity relative to DMS, with the additional sepiolite filter showing the greatest improvement. Thus, a sensor sensitive to 2 ppm DMS (S = 1.7) was obtained; however, in contrast to earlier mentioned articles on chemoresistors, the atmosphere next to the DMS contained 90% relative humidity, since, from the point of view of medical diagnostics, the presence of water vapour in exhaled air can significantly affect VSC detection.

In this article, the authors based on the knowledge gained and described previously present next modifications in terms of developing a DMS sensor with higher sensitivity and lower detection limit to develop sensors suitable for detecting, e.g., halitosis markers in exhaled air. Improved performance was achieved by changing the microstructure of ZnO (from grains to nano- and microplates), increasing the content of gold nanoparticles as a dopant (from 0.25 wt.% to 0.75 wt.%) and using the proven and previously described sepiolite-based passive filter [37]. Modification of the ZnO-based layer composition enabled the development of a sensor highly sensitive to 1 ppm DMS (S = 11.4), achieving an LOD of as much as 406 ppb, despite the presence of additional high water vapour content (90% RH) in the analysed atmosphere.

## 2. Materials and Methods

Zinc oxide was obtained by chemical synthesis. After mixing an aqueous solution of a precursor containing Zn^2+^ ions (ZnCl_2_) and sodium hydroxide (NaOH) and then exposing the solution to a 120 W microwave field (mf), the following reactions took place for 30 min:

(1) $${ZnCl}_{2}+2NaOH\to {Zn{\left(OH\right)}_{2}}_{↓}+2NaCl,$$

(2) $$Zn{\left(OH\right)}_{2}\overset{mf}{\to }ZnO,$$

When the reaction was complete, the temperature of the solution was about 85 °C and a white ZnO precipitate was visible at the bottom of the beaker. The precipitate was washed several times with deionised water and isopropanol and then used to make gas-sensitive layers.

Gold nanoparticles were obtained by chemical reduction of gold ions in the aqueous solution as described [33], with minor modifications. Low molecular weight polyethyleneimine (PEI) with an average molecular weight of 10 kDa and the surfactant Triton^®^ X-100 were used as stabilisers. The prepared solutions were mixed together in such amounts that the final concentration of ions in the Au^3+^ solution was 0.5 mM. Ascorbic acid was added to a solution containing the precursor of gold ions and stabilizers. The reaction produced a ruby-coloured gold colloidal solution with a gold concentration of 100 ppm.

The doping of the gas-sensitive material with gold nanoparticles was performed by mixing. The process involved dispersing hexagonal ZnO plates in isopropanol, and then a colloidal solution of gold nanoparticles was added to the resulting mixture. The content of gold ions in the solution relative to the weight of zinc oxide was 0.75 wt.%. After thorough mixing on a magnetic stirrer, the solution was dried at 80 °C and then annealed in a furnace at 450 °C for 0.5 h to remove organic residues.

The crystal structure of the hexagonal ZnO nano- and microplates was determined by X-ray diffraction using a Malvern Panalytical Empyrean XRD X-ray diffractometer. CuKα radiation was used. Measurements were made by scanning the sample over a range of θ/2θ angle values.

Microstructure studies of the sensor layer were performed using a SEM SU6600 scanning electron microscope (Hitachi, Hitachinaka, Japan) and an HRTEM Tecnai G2 20 X-TWIN high-resolution transmission electron microscope (FEI Company).

The size distribution of the gold nanoparticles was determined by Dynamic Light Scattering (DLS). For this purpose, a Nicomp 380ZLS (Particle Sizing Systems, USA) containing a 633 nm laser with a power of 50 mW was used. The time for a single measurement was 3 min, and the photon-counting frequency of the autocorrelator was approximately 200 kHz. Polymethylmethacrylate (PMMA) cuvettes measuring 40 × 10 × 10 mm were used in the measurements.

**Figure 1:**
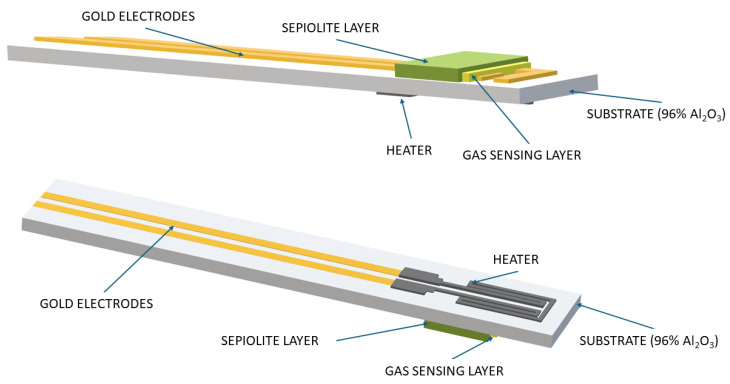
Sensor design (view shows both sides).

For the sensor structure, an alumina ceramic substrate (96% Al_2_O_3_, CeramTec, Plochingen, Germany) with a thickness of 250 µm was used as a structural support element to ensure good mechanical stability. The dimensions of a single sensor support structure were 25.40 × 2.45 × 0.25 mm. The electrodes and heater leads were screen-printed from gold paste (8846-G, ESL Europe, Reading, UK). A meander-shaped platinum heater for uniform temperature distribution on the substrate was made from Pt-5545 paste (ESL Europe, Reading, UK); the platinum meander area was approximately 7.10 mm. The gas-sensitive layer (pure or doped) was made by screen-printing technology on gold electrodes, with a thickness of 40 µm. A sepiolite-based passive filter with a thickness of 40 µm and dimensions of 800 × 1600 µm was also used. The design of the sensors analysed is shown in Figure 1.

Electrical characterisation of the sensors was performed using the temperature-stimulated conductance method (TSC) in a synthetic air atmosphere with a relative humidity of 90% and in a dimethyl sulphide atmosphere with a concentration of 1–10 ppm. The substrate temperature was controlled cyclically and linearly at a constant rate of 2 °/s between 150 °C and 750 °C by using a platinum heater powered by an E3632A DC power supply (Agilent Technologies, Santa Clara, CA, USA) in the design. The current flowing through the gas-sensitive material was recorded during both the rise and fall of temperature. The sensor layer was DC-polarised using a Keithley 2400 current-voltage source (Keithley Instruments Inc., Cleveland, OH, USA). Electrical measurements were recorded using a potentiostat-galvanostat type SI 1287 from Solartron Analytical (Farnborough, UK), using CorrWare 3.5 h software from Scribner Associates Inc. (Southern Pines, NC, USA).

The sensitivities of all developed sensors to DMS were also determined from the temperature changes in conductance of the sensors tested. The sensitivity of the sensors (S) was defined as the ratio of the sensor conductance in the atmosphere containing the gas to be determined (G_gas_) to the conductance in a reference air atmosphere (G_90%air_) with a relative humidity of 90%.

## 3. Results

### 3.1. Characterisation of Materials

**Figure 2:**
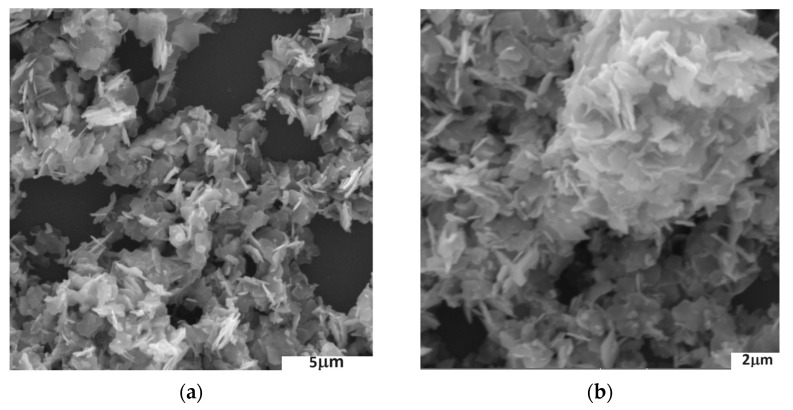
SEM images of obtained ZnO plates: (**a**) under ×13,300 magnification; (**b**) under ×18,000 magnification.

SEM imaging of the resulting zinc oxide showed that exposure of the reaction mixture to a microwave field, and thus an increase in temperature, led to the formation and crystallisation of ZnO in the form of plates (Figure 2a,b).

**Figure 3:**
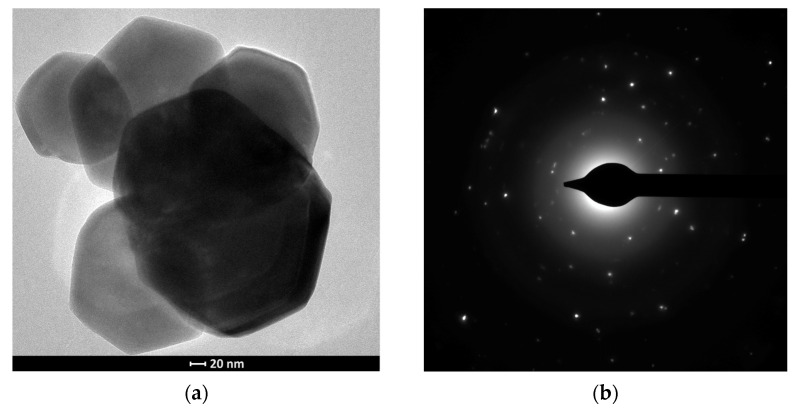
Microstructure of hexagonal ZnO plates: (**a**) TEM image, (**b**) selected area electron diffraction pattern (SAED).

The obtained zinc oxide was characterised by the presence in its structure of hexagonal plates with different diameters of about 1 μm and thicknesses of about 150 nm. TEM analysis of the structures obtained confirmed the hexagonal shape of the crystallites and the polycrystallinity of the material (Figure 3a,b).

**Figure 4:**
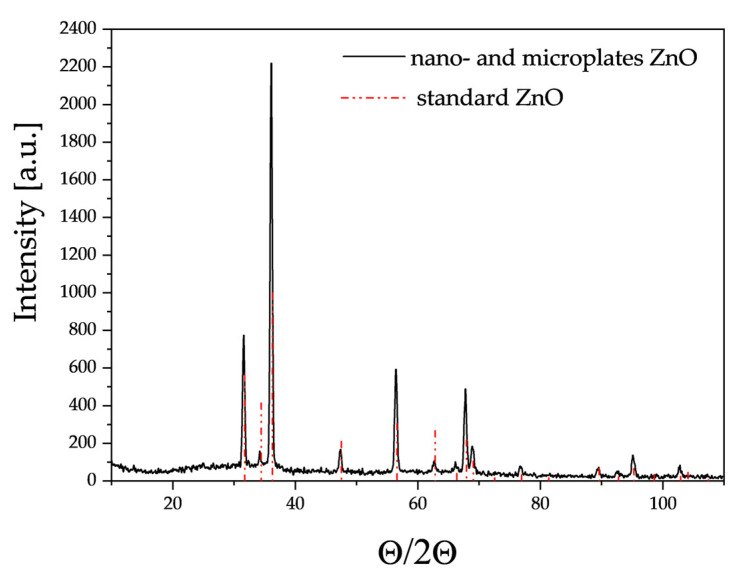
X-ray diffractogram of the resulting zinc oxide nano- and microplates.

XRD studies (Figure 4) showed that the crystallographic structure of the obtained ZnO corresponds to reference zinc oxide with a hexagonal structure, lattice constants a = 3.2501 Å, b = 3.2501 Å and c = 5.2071 Å and space group P63mc.

**Figure 5:**
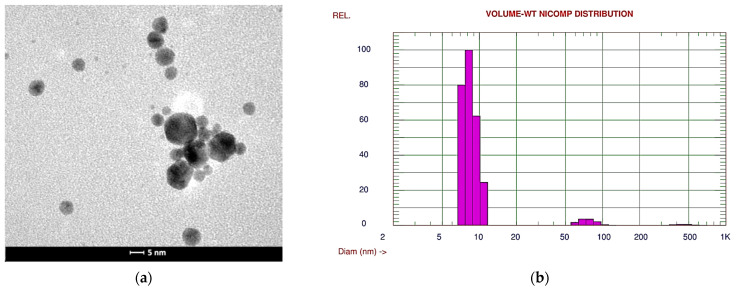
Gold nanoparticles; (**a**) TEM image; (**b**) the size distribution of nanoparticles determined from the volume by weight using DLS technique.

The substructure of the gold nanoparticles was observed using a transmission electron microscope (TEM). Analysis of the TEM images showed that the gold nanoparticles had a spherical shape and the average size of the gold nanoparticles, as determined by dynamic light scattering (DLS), was approximately 13.9 nm (Figure 5a,b).

**Figure 6:**
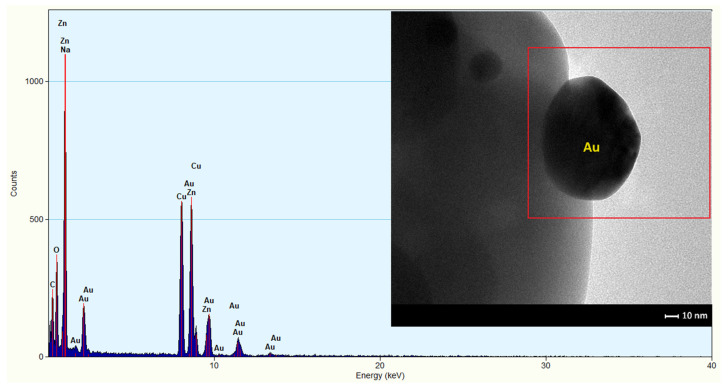
Result of EDS elemental analysis; on the right, a TEM image of gold nanoparticles in doped ZnO.

The hexagonal nano- and microplates were doped by volume with gold nanoparticles at 0.75 wt.%. TEM/EDS analysis of the material confirmed the presence of spherical forms of gold nanoparticles (Figure 6) which were uniformly dispersed on the surface of the material. When ZnO plates were doped, there was no incorporation of gold into the ZnO structure. Au was not visible in the X-ray diffractogram, because the amount of gold was less than 1 wt.% and it was present in very high dispersion.

**Figure 7:**
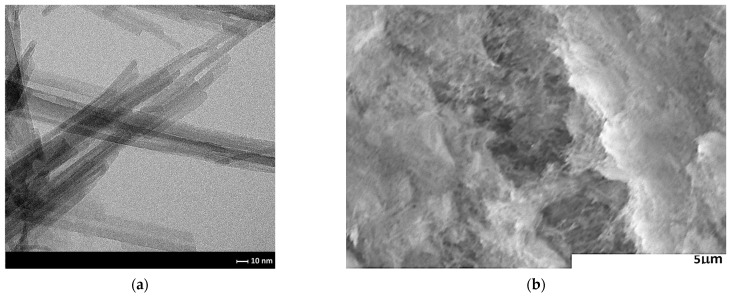
Microstructure of the sepiolite filter; (**a**) TEM image; (**b**) SEM image of the filter surface.

One of the most promising techniques for improving sensor performance is to create sensor structures consisting of at least two layers with different electrical and catalytic properties [38,39]. The layer in direct contact with the electrodes acts as the actual active layer, while other materials, layered on top of it, act as a filter to modify the sensor parameters. Such filters may be characterised as either catalytic (active filters) or non-reactive (passive filters) with respect to the component of the atmosphere to be determined or interfered with. Due to the specific properties of sepiolite, such as high absorption capacity (0.42 cm^3^/g), small channel diameter, large specific surface area (148 m^2^/g at ambient temperature, 263 m^2^/g at 100 °C, 60 m^2^/g at 900 °C), this material was used as a passive filter. Figure 7 shows the surface area of the sepiolite filter before (Figure 7a) and after printing (Figure 7b).

As can be seen, this material is characterised by a developed surface with a fibrous structure, with visible tubes/channels resulting in unique properties. The material analysis and microstructure of sepiolite was comprehensively presented in the authors’ previous article [37].

### 3.2. Electrical Characterisation

The following three types of sensors were fabricated, differing in the composition of the gas-sensitive layer:

- A gas-sensitive layer in the form of hexagonal ZnO nano- and microplates (ZnO plates);
- A gas-sensitive layer in the form of hexagonal ZnO nano- and microplates doped with 0.75 wt.% gold nanoparticles (ZnO plates/Au(vol));
- A gas-sensitive layer in the form of hexagonal ZnO nano- and microplates doped with 0.75 wt.% gold nanoparticles, additionally coated with a sepiolite filter (ZnO plates/Au(vol)/sepiolite).

The sensors were electrically characterised in an atmosphere: with a high water vapour content of 90% RH and with a high humidity of 90% RH containing DMS in the concentration range 1–10 ppm.

**Figure 8:**
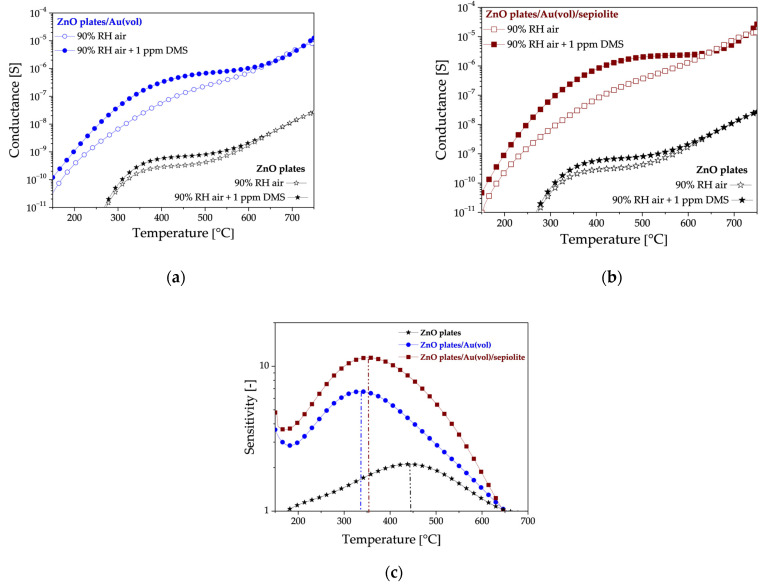
Thermal changes in the (**a**) conductance of ZnO plates/Au(vol) and ZnO plates sensors, (**b**) conductance of ZnO plates/Au(vol)/sepiolite and ZnO plates sensors, (**c**) sensitivity of all sensors in an atmosphere of 90% relative humidity containing 1 ppm DMS. The vertical dashed lines correspond to the temperature at which the maximum sensitivity was recorded for each sensor.

An analysis of the sensor test results in high humidity atmospheres has revealed that all the sensors exhibit a notable response to low DMS concentrations. This is evidenced by the significant increase in their conductance in the presence of DMS compared to their initial conductance (Figure 8a,b). Doping with gold nanoparticles increased the sensitivity to the sulphur marker. It should be noted that doping with gold nanoparticles resulted in a shift of the sensitivity maximum towards the lower temperature (Figure 8c). The highest sensitivity was obtained when the doped layer was covered with a sepiolite filter.

**Figure 9:**
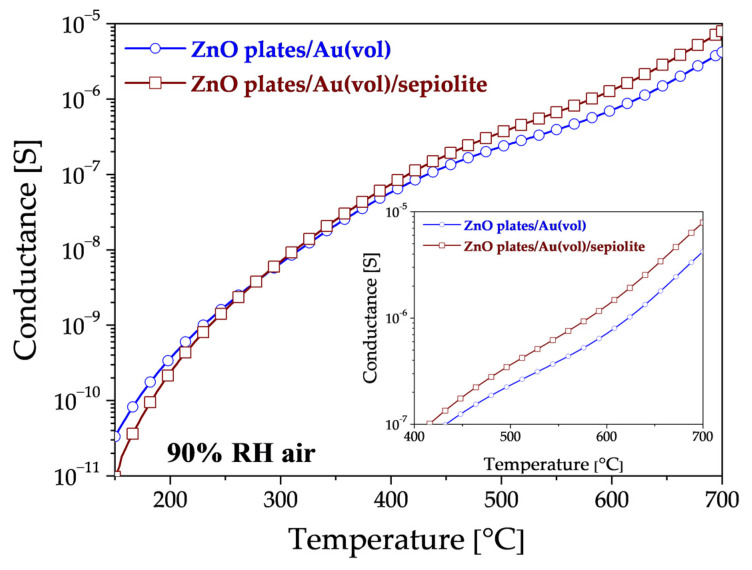
Changes in the conductance of ZnO plates/Au(vol) and ZnO plates/Au(vol)/sepiolite in 90% RH air, without DMS. The smaller graph shows a close-up view of the high-temperature area.

Upon analysis of the impact of sepiolite on sensor conductivity in a high-humidity environment (without DMS), three distinct ranges of areas emerge (Figure 9). In the low-temperature range up to approximately 280 °C, the conductance of the sensor with a sepiolite layer is observed to be lower than that of the sensor without sepiolite (Figure 9). This is due to the fact that water molecules from the environment are adsorbed, thereby preventing adsorption on the surface of the sensor material. In the second range of 280–380 °C, the conductance of both sensors is similar, indicating that water desorbs from both the surface and bulk of the sepiolite and ZnO. In the range above 380 °C, the conductivity of the sensor with a sepiolite filter is higher than that of the sensor without a filter, suggesting that the release of water molecules from the nodes of the octahedral lattice system in sepiolite may be a contributing factor [40,41].

**Figure 10:**
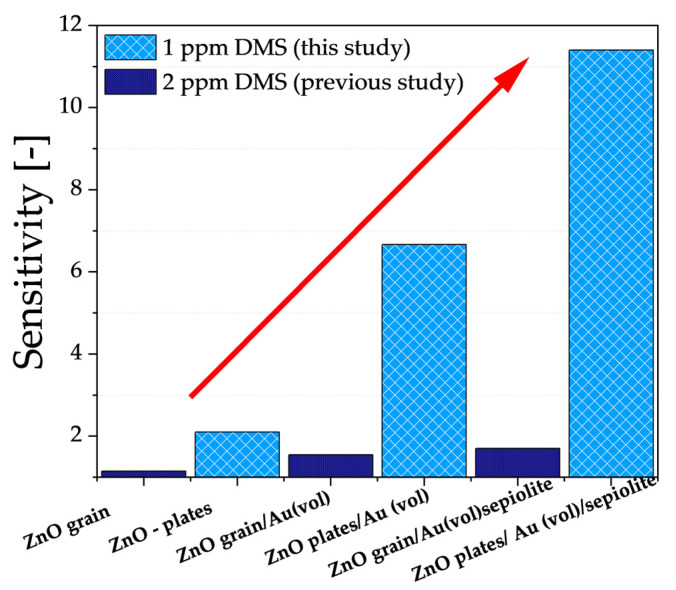
Sensitivity of the developed sensors in an atmosphere containing 1 ppm DMS at 90% relative humidity with respect to the authors’ previous results, where the lowest DMS concentration analysed was 2 ppm [21]. Sensitivity determined at the temperature at which the sensor showed the greatest change in conductance.

As previously mentioned, the results described in this paper are a continuation of research into improving the properties of gas-sensitive resistive sensors relative to DMS, where the active layer is ZnO doped with gold nanoparticles and a sepiolite filter is used. It was crucial that measurements were also performed in a high-humidity atmosphere. Based on previous studies, the authors undertook to modify the microstructure of ZnO (from grains to hexagonal nano- and microplates) and to increase the content of gold nanoparticles (from 0.25 wt.% to 0.75 wt.%). Based on the electrical characterisation of all sensors, there was a significant improvement in the sensitivity of all sensors (Figure 10).

As can be seen, the modification of ZnO microstructure contributed to an increase in the specific surface area interacting with the sulphur marker, thereby enhancing sensor sensitivity (ZnO grain → ZnO nano- and microplates). Furthermore, by increasing the amount of nanogold (0.25 wt.% → 0.75 wt.%) with high affinity to sulphur and high dispersion in the volume of the sensor material, the DMS-detection capability of the developed sensor was improved four times despite the lower DMS concentration than analysed in previous studies. Finally, the combination of both modifications and the use of a proven sepiolite filter resulted in a notable enhancement in sensitivity to 1 ppm DMS, despite the high-humidity environment.

**Figure 11:**
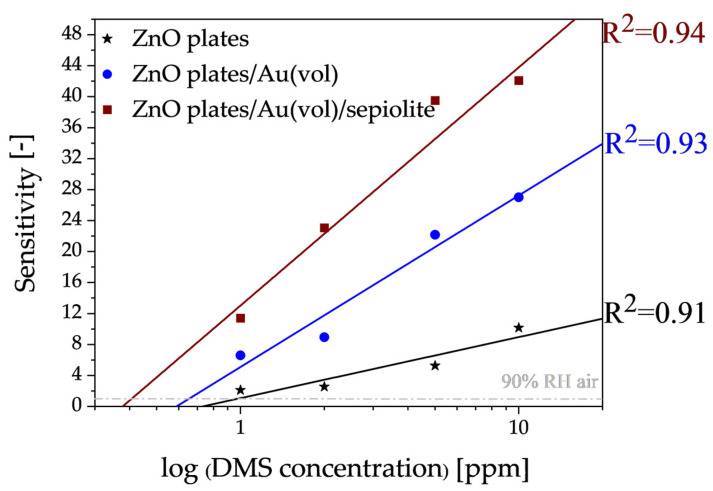
Approximation of the lower detection limits of the developed sensors for dimethyl sulphide in an atmosphere with a relative humidity of 90%.

Another important parameter of the aforementioned sensors, from the point of view of halitosis diagnosis, besides sensitivity, is also the lower detection limit. The dependence of sensitivity on the concentration of any gas can be defined by the following relation:

(3) $$S=p^{n}$$

where *p* is a partial pressure of gas. The value of the exponent *n* depends on the type of sensor material, its microstructure and the type of gas to be detected. In a narrow range of concentrations, this exponential relationship can be approximated by a linear relationship. As shown in Figure 11, there is a good linearity between the DMS concentrations and the sensitivity values and the linear correlation coefficient was very high (0.91–0.94). Thus, the detection limit in this case was determined from the intersection of the trend line with the line for which the sensitivity is equal to 1 (Figure 11).

It is desirable for halitosis marker sensors to have the lowest detection limit. On the basis of the experiments carried out, it was found that a layer enabling the detection of dimethyl sulphide at a level of approximately 406 ± 6 ppb could be made with a ZnO plates/Au(vol)/sepiolite sensor. In addition to the fact that the sensor allowed detection in aggressive environments (sulphur, humidity), the use of cyclic layer annealing after the measurements allowed for complete desorption of the measurement residue and reuse of the sensor.

## 4. Discussion

The change in conductivity of the gas-sensitive layer in the presence of dimethyl sulphide is caused by catalytic oxidation reactions of this gas occurring on the surface of the gas-sensitive material. The mechanism of oxidation of dimethyl sulphide is complex, but schematically it can be described by the following sum reaction, which is the result of a series of reactions [20,42]:

(4) $${CH}_{3}SCH_{3}+9O^{-}\to {2{CO}_{2}+3H}_{2}O+{SO}_{2}+9e^{-}$$

This process depends on the temperature, the type of sensor material, its microstructure as well as the dopants used. Since it is an oxidation reaction, the adsorption of oxygen on the surface defects of the oxide semiconductor crystal lattice is therefore crucial [26]. In the case of the analysed hexagonal nano- and microplates, zinc oxide, the bulk and surface parameters of this material are closely correlated with the point defects present such as zinc interstice, oxygen interstice, zinc atoms in interstitial sites, oxygen atoms in interstitial sites, zinc atoms in place of oxygen and oxygen atoms in place of zinc. There are also combinations of defects in this oxide, e.g., Schottky pair (anion and cation interstice) and Frenkel pairs (cation gap and interstitial cation) [33]. The dominant defects in ZnO may be oxygen vacancies, causing an increase in the concentration of donor levels, although the literature also reports a dominant role for defects in the form of zinc located in the interstitial position [43,44]. These defects are the result of processes occurring during the thermal treatment of ZnO and result in the formation of numerous donor and acceptor levels in the forbidden gap.

**Figure 12:**
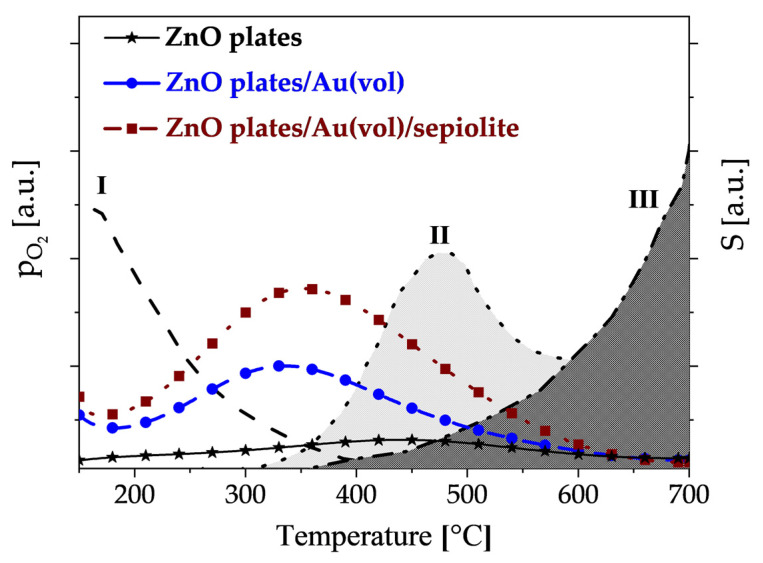
Variation of oxygen pressure above the ZnO surface as a function of temperature and sensor sensitivity to DMS in a 90% RH atmosphere (with reference to [45]); I—chemisorption of oxygen coupled with charge exchange with ZnO; II—desorption of chemisorbed oxygen bound to the ZnO surface; III—sublimation of highly defected zinc oxide.

Comparing the temperature changes in the sensitivity of individual sensors towards DMS with the temperature changes in oxygen pressure [45] above the ZnO surface (Figure 12), it can be seen that if the atmosphere, in addition to water vapour, contains DMS, oxidation of this compound occurs intensively in the region when physically adsorbed oxygen is removed from the surface and chemical adsorption of oxygen begins.

The oxidation of DMS occurs intensively on centres not directly involved in the competitive process, i.e., the filling of active centres with oxygen molecules. The maximum sensitivity to DMS of gold-doped sensors is shown at around 350 °C. The exception is undoped zinc oxide, which only shows maximum sensitivity at about 450 °C, i.e., when zinc oxide-sublimation begins. This demonstrates the lower activity of zinc oxide towards sulphur compounds.

It is widely acknowledged that the structure of the gas-sensitive material is a significant factor influencing the interaction mechanism of the sensor material with gases, such as dimethyl sulphide. Zinc oxide in the form of ZnO nano- and microplates compared to previously studied ZnO grains [21] is characterised by a higher surface area to volume ratio, a higher rate of electron and hole diffusion to the surface of nano-sized structures and surface anisotropy, which is important during chemical sorption of gases [36]. The results clearly demonstrated that during DMS detection, the change of structure from grains to nanoplates results in a significant improvement in sensitivity.

**Figure 13:**
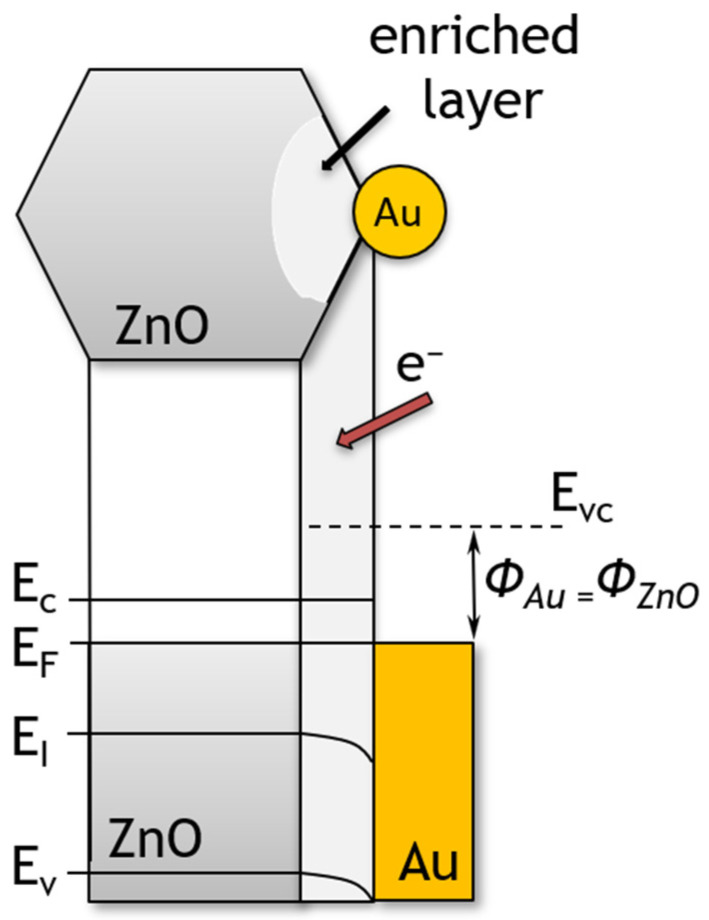
The structure of energy band of ZnO hexagonal nano- and microplates modified with Au nanoparticles. *E_VC_*, *E_C_*, *E_F_*, *E_I_*, *E_V_* and *Φ* denote the vacuum energy, conduction band minimum, Fermi level, Fermi level for intrinsic semiconductor, valence band maximum and work function, respectively.

Another important factor influencing sensor performance is the doping of the sensor material. The addition of gold nanoparticles, a noble metal with a high chemical affinity to sulphur, resulted in a fourfold increase in sensitivity to sulphide compared to an undoped layer, as evidenced in the literature [46,47,48]. A distinction can be made between dopants that cause changes in, e.g., degree of crystallinity of the sensing layer [49] or affecting the kinetics of reactions occurring on the surface of the sensor material—referred to as promoters [50]. Improvements in the detection capability of VSCs can be achieved by two mechanisms: electrical or chemical. In the electro-mechanism, doping results in the formation of a zinc oxide material with gold nanoparticles on the surface (Figure 6). The formation of Schottky metal/semiconductor barriers between metallic Au islands and zinc oxide, which exhibits semiconducting properties, is therefore to be expected. It is known that the work function of zinc oxide nanostructures is approximately 5.2–5.3 eV [51,52], while that of gold is 4.68–5.1 eV [53,54]. It can be seen, therefore, that a Schottky barrier is formed at the interface between the zinc oxide and the gold. Since the Fermi levels of Au and ZnO must be balanced, the electrons pass from gold into ZnO (Figure 13) [55]. Thus, Au nanoparticles cause local changes in the Fermi level of zinc oxide. As a result of this process, Au islands become positively charged and an electron-enriched layer is formed near these islands on the surface of ZnO particles [33]. The increase in electron concentration results in an increase in the amount of oxygen chemisorbed on the surface of the sensor material [26]. It was thus observed that the conductance of ZnO nano- and microplates that had been modified with Au nanoparticles was higher than that of the unmodified ZnO.

**Figure 14:**
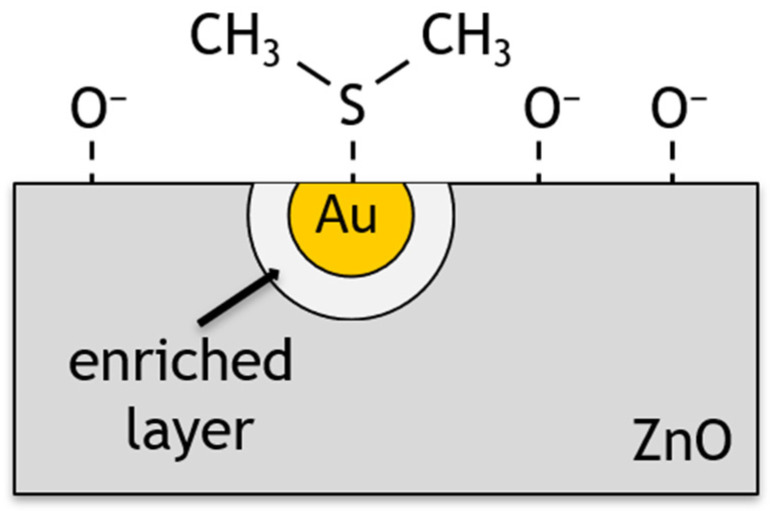
Schematic representation of ZnO surface doped with gold nanoparticles during DMS detection.

In the chemical mechanism, the dopant is treated as an activator of the sensor layer to catalytically promote chemisorption. Au is also known to promote the catalytic dissociation of molecular oxygen species which is also known as spillover in catalysis [56,57]. An increase in the concentration of chemisorbed oxygen on the surface causes a process of oxygen spillover to the ZnO surface, where oxygen is involved in surface reactions (Figure 14). These processes result in a change in the surface energy of the material and its resistivity, while the chemical composition of the dopant remains unchanged [58,59].

**Figure 15:**
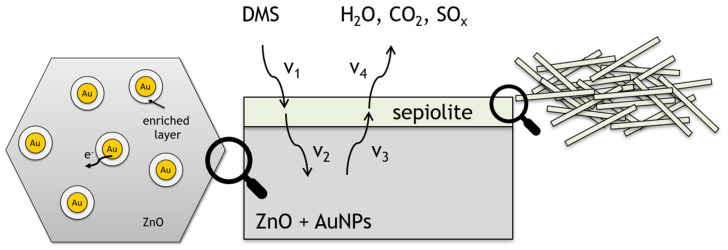
Schematic representation of ZnO surface doped with gold nanoparticles with sepiolite filter during DMS detection.

The last of the proposed modifications involved the use of a sepiolite filter. Sepiolite is a dielectric, and thus no potential barrier is formed at the gas-sensitive layer–sepiolite interface. Furthermore, is also not catalytically active, so the sepiolite layer should be considered as a diffusion barrier not only for gas molecules diffusing into the sensing material, but also affecting the desorption process of reaction products. The layer will have the greatest effect on molecules with a large dipole moment. Assuming complete oxidation of dimethyl sulphide (4), water is released on the surface of the sensor layer. Consequently, sepiolite will have the greatest impact on the diffusion of water formed during the oxidation of the sulphur markers (Figure 15). Moreover, water molecules are present in the ambient atmosphere (high humidity) and are embedded in the sepiolite structure. Water released from the sepiolite at different temperature ranges diffuses not only into the ambient atmosphere, but also into the gas-sensitive material, causing a change in its conductivity. In accordance with the findings of the authors of reference [37], the rate of diffusion of reactants and products from the atmosphere to the surface of the sensor and vice versa is identical (V_1_, V_4_). The filter affects the rate of diffusion, adsorption and desorption of reactants and products (V_2_, V_3_) within a filter (Figure 15).

Carbon dioxide molecules are also formed as a result of the DMS oxidation reaction. Their interaction with the filter layer should be neglected as they are molecules without a dipole moment. They can interact with adsorbed water molecules, but in a temperature range close to the ambient temperature [37].

It can be concluded that the obtained sensors allow the detection of dimethyl sulphide at low concentrations in the presence of high humidity, which is important for the detection of markers present in exhaled air. In the authors’ opinion, in the case of dimethyl sulphide, it is necessary to consider next modifications to the composition of the sensor layer in order to further reduce the detection limit. The direction of the proposed changes is appropriate and worthy of further consideration.

## Data Availability

The data are available from the corresponding author upon request.
